# Responses to the Gaza-Israel Conflict by Specialty Medical Societies

**DOI:** 10.1001/jamanetworkopen.2025.4662

**Published:** 2025-04-11

**Authors:** Manasa S. Pagadala, Natasha Nichols

**Affiliations:** 1 Northwestern University Feinberg School of Medicine, Chicago, Illinois

## Abstract

This cross-sectional study evaluates the statements and actions of medical and surgical societies recognized by the Council of Medical Specialty Societies regarding the Gaza-Israel conflict.

## Introduction

The Gaza-Israel conflict has caused catastrophic damage to health care infrastructure, with hospitals, clinics, and medical supply chains severely disrupted. Reports from organizations such as the United Nations, World Health Organization, and International Committee of the Red Cross highlight the devastating impact on critical health care services.^1,2,3^ This crisis underscores the need for a coordinated response to address both immediate and long-term health challenges, including mental health care, infectious disease control, and maternal and child health services.

Medical societies recognized by the Council of Medical Specialty Societies (CMSS) (Box) are in a unique position to lead during response to such crises. Historically, medical societies not only set care standards for the profession, but also play a pivotal role in addressing global humanitarian emergencies by raising awareness, advocating for affected populations, organizing medical mission work, and providing resources and training for local health care professionals.^4,5^ Examples include their responses to conflicts in Syria, natural disasters like the 2010 Haiti earthquake, and the COVID-19 pandemic, where they mobilized expertise, supplies, and educational initiatives.^4,5^ This study examines the statements and actions of CMSS-recognized medical and surgical societies regarding the Gaza-Israel conflict and which organizations have used their platforms to address the crisis.

Box. **List of Societies Formally Recognized by the Council of Medical Specialty Societies Included in the Analysis**

American Academy of Allergy, Asthma, and Immunology
American Academy of Dermatology
American Academy of Family Physicians
American Academy of Hospice and Palliative Medicine
American Academy of Neurology
American Academy of Ophthalmology
American Academy of Orthopaedic Surgeons
American Academy of Pediatrics
American Academy of Physical Medicine and Rehabilitation
American Association for the Study of Liver Diseases
American Association of Clinical Endocrinology
American College of Cardiology
American College of Chest Physicians
American College of Emergency Physicians
American College of Medical Genetics and Genomics
American College of Obstetricians and Gynecologists
American College of Occupational and Environmental Medicine
American College of Physicians
American College of Preventive Medicine
American College of Radiology
American College of Rheumatology
American College of Surgeons
American Epilepsy Society
American Gastroenterological Association
American Geriatrics Society
American Medical Informatics Association
American Psychiatric Association
American Society of Addiction Medicine
American Society of Anesthesiologists
American Society of Clinical Oncology
American Society for Clinical Pathology
American Society of Breast Surgeons
American Society of Colon and Rectal Surgeons
American Society of Hematology
American Society of Nephrology
American Society of Plastic Surgeons
American Society for Gastrointestinal Endoscopy
American Society for Radiation Oncology
American Society for Reproductive Medicine
American Thoracic Society
American Urological Association
College of American Pathologists
Infectious Diseases Society of America
North American Spine Society
Obesity Medicine Association
Society for Post-Acute and Long-Term Care Medicine
Society for Vascular Surgery
Society of Critical Care Medicine
Society of General Internal Medicine
Society of Gynecologic Oncology
Society of Hospital Medicine

## Methods

For this cross-sectional study, *statement* refers to any formal acknowledgment on the organizations’ official websites. We analyzed public statements and resources released by CMSS-recognized societies from November 1, 2023, to July 10, 2024. Informed consent and institutional review board approval were not required in accordance with 45 CFR §46 because the study used publicly available data. This study adheres to the Strengthening the Reporting of Observational Studies in Epidemiology (STROBE) reporting guidelines.

We searched the organizations’ websites for hyperlinks that included the terms *statement*, *Gaza*, *Israel*, *Palestine*, *conflict*, *resources*, and *volunteers*. This search was also applied to their partner journals, where the journals’ online archives and recent publications were reviewed. Each organization’s published statements, publication in partner journals, resources provided, calls for volunteers, and outreach efforts were recorded.

Primary end points included the presence of official statements regarding the Gaza-Israel conflict. Secondary end points included publications regarding the conflict in partner journals, provision of resources, and any calls for volunteers or outreach efforts.

## Results

Of the 53 medical and surgical societies included in the analysis, 13 societies (24.5%) had statements regarding the Gaza-Israel conflict on their websites, while 40 societies (75.5%) did not. Four societies (7.5%) had publications regarding the conflict in their partner journals. Five societies (9.4%) shared information about organizations in Israel and Palestine to which people can donate. One society (1.9%) issued a call for volunteers, and 1 society (1.9%) provided resources to medical practitioners in the region (Figure).

**Figure.**
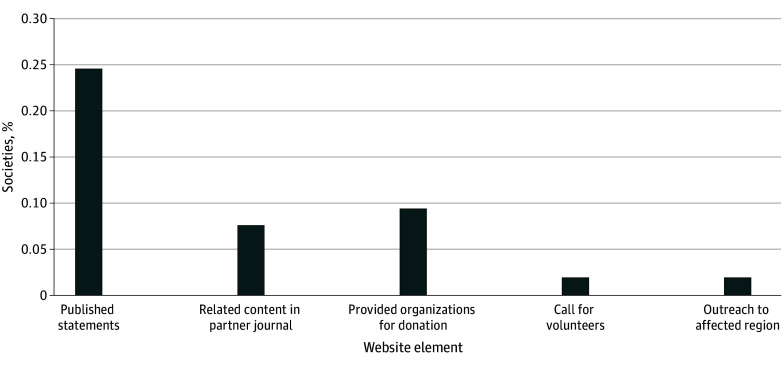
Percentage of the 53 Council of Medical Specialty Societies–Recognized Societies Displaying Each Element on Their Website

## Discussion

Given the historical role of medical societies during a crisis, the findings from this cross-sectional study suggest an ongoing need to leverage their positions. The Gaza-Israel conflict represents an ongoing health crisis with profound implications for health care systems, health care professionals, and vulnerable populations. Although we are limited due to a lack of access to CMSS-recognized societies’ private website communications to members, our study revealed that 24.5% of societies issued a public statement on the Gaza-Israel conflict. Medical societies have historically demonstrated their ability to advocate for health equity during conflicts and humanitarian emergencies, such as the war in Ukraine, the crises in Myanmar, and the aftermath of the Iraq invasion.^6^ The study is limited in that it focuses on a single conflict and does not have comparative data of medical specialty society statements for other conflicts. In addition, the direct effect of statements or actions to mitigate the devastating health impacts of armed conflict is unknown and deserves further research.

International frameworks, such as the World Medical Association’s Declaration of Geneva, emphasize the role of the medical community in safeguarding health and dignity during crises. By engaging in advocacy, education, and resource mobilization, medical societies can play a pivotal role in addressing the immediate and long-term consequences of war on health systems.

## Notes

Supplement.
Data Sharing Statement
