# Supplementary material for: Responses to the Gaza-Israel Conflict by Specialty Medical Societies
Source: JAMA Netw Open. 2025 Apr 11;8(4):e254662. doi: 10.1001/jamanetworkopen.2025.4662 (PMC11992601; doi:10.1001/jamanetworkopen.2025.4662)
Supplement: Supplement. — Data Sharing Statement [file jamanetwopen-e254662-s001.pdf]

Data Sharing Statement

Pagadala. Responses to the Gaza-Israel Conflict by National Medical Societies. *JAMA Netw Open*. Published April 11, 2025. doi:10.1001/jamanetworkopen.2025.4662

Data
Data available: No

Additional Information
Explanation for why data not available: It can be found on publicly available websites.
